# Developmental outcomes in children exposed to Zika virus in utero from a Brazilian urban slum cohort study

**DOI:** 10.1371/journal.pntd.0009162

**Published:** 2021-02-05

**Authors:** Juan P. Aguilar Ticona, Nivison Nery, Joseph B. Ladines-Lim, Claudia Gambrah, Gielson Sacramento, Bruno de Paula Freitas, Joseane Bouzon, Jamary Oliveira-Filho, Ana Borja, Haritha Adhikarla, Magelda Montoya, Athena Chin, Elsio A. Wunder, Verena Ballalai, Carina Vieira, Rubens Belfort, Antonio R. P. Almeida, Mitermayer G. Reis, Eva Harris, Albert I. Ko, Federico Costa

**Affiliations:** 1 Instituto de Saúde Coletiva, Universidade Federal da Bahia, Salvador, Brazil; 2 Instituto Gonçalo Moniz, Fundação Oswaldo Cruz,Ministério da Saúde, Salvador, Brazil; 3 Department of Epidemiology of Microbial Diseases, Yale School of Public Health, New Haven, Connecticut, United States of America; 4 Hospital Geral Roberto Santos (HGRS), Salvador, Brazil; 5 Faculdade de Medicina, Universidade Federal de São Paulo, São Paulo, Brazil; 6 Faculdade de Medicina da Bahia, Universidade Federal da Bahia, Salvador, Brazil; 7 Programa de Pòs-Graduação em Ciencias da Saude (PPgCS) Universidade Federal da Bahia, Salvador, Brazil; 8 Departamento de Fonoaudiologia. Instituto de Ciências da Saúde. Universidade Federal da Bahia, Salvador, Brazil; 9 Division of Infectious Diseases and Vaccinology, School of Public Health, University of California, Berkeley, California, United States of America; Federal University of Ceará, Fortaleza, Brazil, BRAZIL

## Abstract

**Background:**

The prevalence of developmental alterations associated with in-utero Zika virus (ZIKV) exposure in children is not well understood. Furthermore, estimation of the Population Attributable Fraction (PAF) of developmental alterations attributed to ZIKV has not been performed due to lack of population-based cohorts with data on symptomatic and asymptomatic ZIKV exposures and an appropriate control group. The aim of this study was to characterize neurodevelopmental outcomes of children at 11 to 32 months of age with intrauterine ZIKV exposure and estimate the PAF of alterations secondary to ZIKV exposure.

**Methodology/Principal findings:**

We performed a cohort of biannual community-based prospective serosurveys in a slum community in Salvador, Brazil. We recruited women participating in our cohort, with a documented pregnancy from January 2015 to December 2016 and children born to those mothers. Children were classified as ZIKV exposed in utero (born from women with ZIKV seroconversion during pregnancy) or unexposed (born from women without ZIKV seroconversion or that seroconverted before/after pregnancy) by using an IgG monoclonal antibody blockade-of-binding (BoB). We interviewed mothers and performed anthropometric, audiometric, ophthalmological, neurologic, and neurodevelopmental evaluations of their children at 11 to 32 months of age. Among the 655 women participating in the cohort, 66 (10%) were pregnant during the study period. 46 (70%) of them completed follow-up, of whom ZIKV seroconversion occurred before, during, and after pregnancy in 25 (54%), 13 (28%), and 1 (2%), respectively. The rest of women, 7 (21.2%), did not present ZIKV seroconversion. At 11 to 32 months of life, the 13 ZIKV-exposed children had increased risk of mild cognitive delay (RR 5.1; 95%CI 1.1–24.4) compared with the 33 children unexposed, with a PAF of 53.5%. Exposed children also had increased risk of altered auditory behavior (RR 6.0; 95%CI 1.3–26.9), with a PAF of 59.5%.

**Conclusions:**

A significant proportion of children exposed in utero to ZIKV developed mild cognitive delay and auditory behavioral abnormalities even in the absence of gross birth defects such as microcephaly and other neurodevelopmental domains. Furthermore, our findings suggest that over half of these abnormalities could be attributed to intrauterine ZIKV exposure.

## Author summary

ZIKV is a neurotropic virus associated with congenital abnormalities that have been grouped under congenital Zika syndrome (CZS), the most prominent being microcephaly. Recent studies have uncovered a spectrum of other abnormalities. However, what remains unclear is the Population Attributable Fraction (PAF) of developmental alterations attributable to ZIKV intrauterine exposure in children (> one year of life). In this population-based cohort study, we found that children (without microcephaly) exposed in utero to ZIKV have an increased incidence of mild cognitive delay and auditory behavior abnormalities, with over half of these events attributable to intrauterine exposure. The results of this study suggest that more than half of alterations found in the population study can be attributed to intrauterine ZIKV exposure, thus demonstrating the importance of monitoring apparently healthy children born during the epidemic, even to asymptomatic mothers. Health services should implement early interventions to limit the morbidity of congenital ZIKV infection.

## Introduction

Intrauterine Zika virus (ZIKV) infection can lead to teratogenic effects, including microcephaly, grouped under the term congenital Zika syndrome (CZS) [1,2]. However, the possibility of development of other manifestations, such as epilepsy and neurodevelopmental abnormalities, is currently under investigation [1,3,4]. While severe outcomes of CZS have been extensively described, limited prospective information exists regarding the effects of congenital ZIKV infection on the neurodevelopmental outcomes of children, particularly those without apparent defects at birth [5]. Similar to other neurotropic viruses, ZIKV may cause subtle alterations that go undetected until later in life [6–8]. One study in the U.S. Territories and Freely Associated States reported that 9% of their pediatric study population had at least one neurodevelopmental abnormality associated with congenital ZIKV infection [3]. Similar studies that followed children without microcephaly in Brazil [9–11] found that children may develop mild or even severe cognitive delay. In contrast, other cohorts in Colombia [12] and the United States [13] did not find severe sequelae in children exposed to ZIKV in utero and born without microcephaly but still emphasize the need for follow-up beyond one year of life.

The reasons for these contrasting results could be due to differences in study settings or other unknown factors. Furthermore, these studies have primarily focused on mothers with symptomatic infection, and most have also focused on children with birth defects, notably microcephaly. This has prevented estimation of the population attributable fraction (PAF) of neurodevelopmental abnormalities secondary to intrauterine ZIKV exposure, regardless of maternal symptomatology and presence of defects at birth. Characterizing the spectrum and incidence of neurodevelopmental abnormalities related to congenital ZIKV infection, in children born to both symptomatic and asymptomatic mothers, could lead to a better understanding of the clinical sequelae of CZS and consequently more timely interventions and improved outcomes. In this study, we prospectively characterized development of children at 11 to 32 months of age exposed to ZIKV in utero in a slum community in Salvador, Brazil and estimated the PAF of abnormalities secondary to intrauterine ZIKV exposure.

## Methods

### Ethics statement

This study was approved by the Institutional Review Boards of Yale University (1006006956) and the ethics committee of the Hospital Geral Roberto Santos–Bahia (1.866.918). All caregivers provided signed consent for interviews, blood collection, and clinical evaluations.

### Study site

This study was performed in Salvador, one of the epicenters of the Zika epidemic [14]. Our study area was the slum community of Pau da Lima, where 73% (95% CI 70%-76%) of individuals were infected by ZIKV during the 2015 epidemic [14]. We identified pregnant women during the epidemic, with and without seroconversion to ZIKV, and performed neurodevelopmental and anthropometric evaluations of their children at 11 to 32 months of age.

This work built upon a long-term prospective cohort study of slum residents that started in 2003, originally focused on Leptospirosis [15,16]. Our last study census from 2013 identified a high population density (14,122 individuals in 3,689 households in 0.17 km^2^). This slum community notably has significant socioeconomic determinants, such as low median income (US$ 1.30 per capita per day per household), the presence of illegal settlements, and substandard sanitation [16].

**Fig 1:**
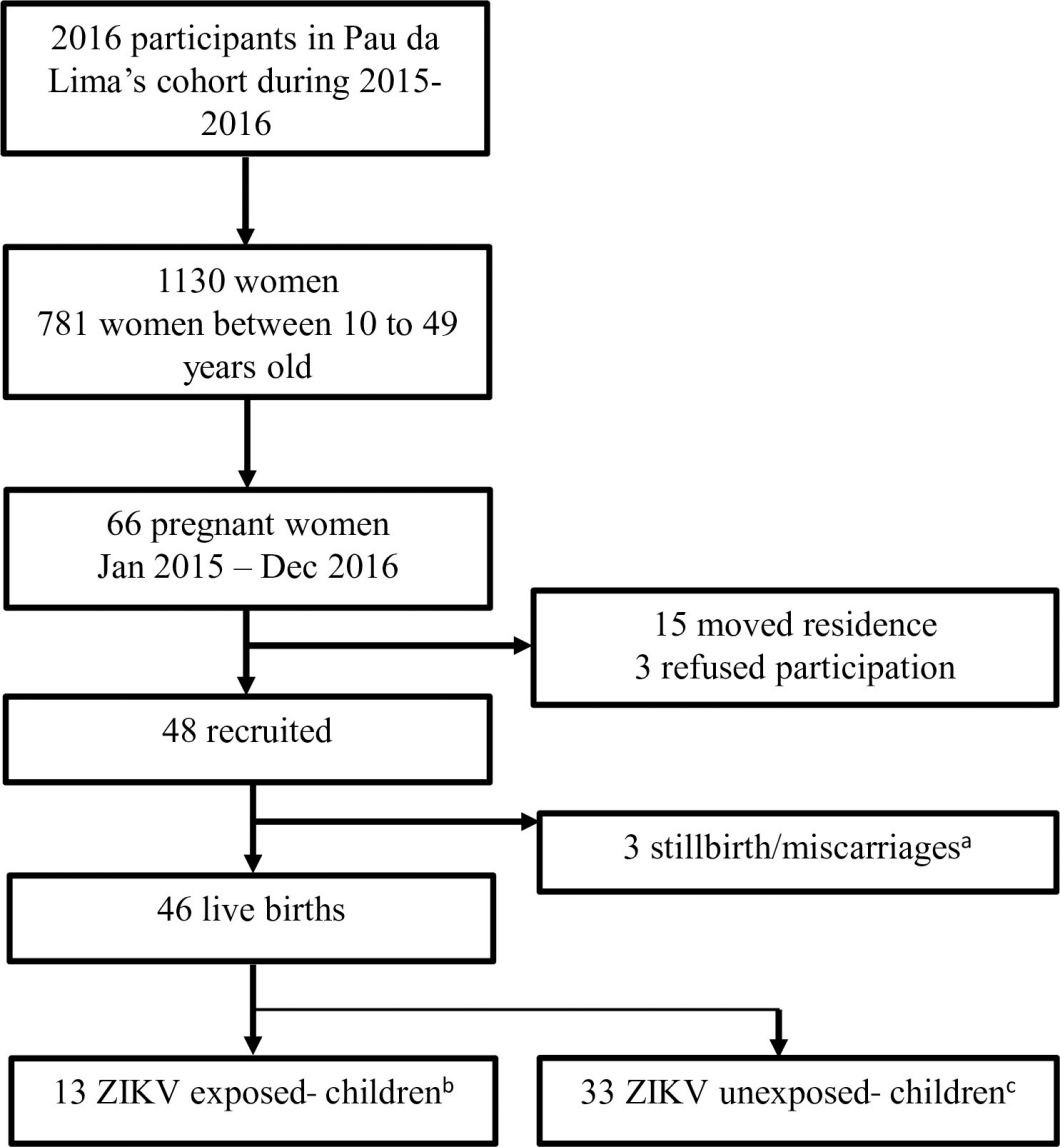
Study flowchart. * One participant reported two pregnancies during the study period, one ending in stillbirth due to extreme premature delivery at 26 weeks and the other ending in a live singleton birth. The live birth was included in the 46 live births that were followed. † ZIKV-exposed children born to mothers with seroconversion to ZIKV during pregnancy.‡ ZIKV-unexposed children born to mothers who had not been infected during pregnancy (without ZIKV seroconversion or ZIKV seroconversion before or after pregnancy).

Using follow-up data from 2013 as a baseline population, we randomly selected 3,716 participants ≥5 years old. Of these, 2,421 (65.1%) fulfilled the inclusion criteria of the study by providing informed consent and spending >3 nights per week in a household inside the study area. Of these, we followed 2016 (83.3%) participants in serologic surveys performed twice annually (August to September and February to March), in both 2015 and 2016. Among participants we followed, 781 (38.7%) were women between 10 and 49 years old in 2016. Of these, women who reported pregnancy from January 1st, 2015 to December 31st, 2016 and their newborns were selected for further analysis (Fig 1).

### Intrauterine ZIKV exposure

We used a ZIKV NS1 blockade-of-binding (BOB) ELISA to evaluate mothers’ exposure to ZIKV in all women included in this study [17,18]. This assay has a reported sensitivity and specificity of 92.0–95.0% and 89.0–95.9%, respectively, and can distinguish ZIKV infections from dengue virus infections [17,18]. We classified a positive ZIKV response using a cut-off of ≥50% inhibition in the BOB value: {1 –[(OD sample–OD minimum value)/delta value]} x 100, where OD represents optical density [17]. We defined intrauterine ZIKV exposure as seroconversion between consecutive serosurveys during pregnancy in the epidemic period. We divided intrauterine ZIKV exposure into two groups: a) “confirmed” when two samples were available during pregnancy, the first being negative and the second being positive, and b) “probable” when one sample during pregnancy was negative and the second obtained after delivery (~4 months) was positive. For probable cases, ZIKV infection may have occurred during pregnancy or after delivery. Lack of exposure was defined as absence of seroconversion or seroconversion that occurred before (mean of 3.6 months) or after pregnancy. We estimated the pregnancy period using date of birth and gestational age.

### Outcomes

A multidisciplinary team (physiotherapists, speech therapists, physicians including an ophthalmologist and neurologist, and nurses) collected information on mothers and children using interviews, chart review, and clinical evaluations. We interviewed mothers using a standardized questionnaire to obtain sociodemographic characteristics and medical history, including history of childbirth and the relevant perinatal period. We collected information on each child at birth and during the follow-up period (11 to 32 months of age). For birth information, we reviewed the prenatal Brazilian monitoring program card and Child Health Record during inpatient care to obtain information on gestational age and anthropometric measurements. We performed a comprehensive assessment during the follow up period, collecting growth parameters and evaluating neurodevelopmental, auditory, and ophthalmological outcomes. The multidisciplinary team collecting this information was kept unaware of (blinded to) participants’ serological status.

### Growth parameters

We recorded weight, length, and head circumference at birth (collected from the Child Health Record) and at the time of the study visit. We analyzed these using reference values (Z-scores) from INTERGROWTH-21 [19] and the WHO [20] for measurements collected at birth and during the follow-up period, respectively.

### Neurodevelopmental evaluation

We used the Bayley Scales of Infant and Toddler Development, 3^rd^ ed. (Bayley III), a neurodevelopmental tool validated in Brazil for children between 16 days and 42 months of age. It includes five domains: Cognitive, Language, Motor, Socio-emotional, and Adaptive Behavior. Children can be classified as having severe neurodevelopmental delay (composite score ≤70 [≤-2 SD]), mild delay (71 to 85 [-2 SD to -1 SD]), or normal development (>85) [11,21]. We also used a screening version of the Bayley III tool that focuses on the Cognitive, Language, and Motor domains. The scales are adjusted for the child’s age and categorized into 3 categories: at risk, emerging, and competent. Children classified as at risk and emerging in the Bayley screening were reevaluated using the complete Bayley protocol. Children classified as competent in the Bayley screening were considered > -1 SD in the Cognitive, Language, and Motor domains of the complete Bayley protocol (i.e. normal development) and were pooled with those who underwent the complete Bayley protocol in the final analysis.

We also used the Hammersmith Infant Neurological Examination (HINE) [22], a standardized evaluation for children between 2 and 24 months of age, to categorize severity of neurological deficits. It includes 26 items that evaluate cranial nerve functions, posture, quality and quantity of movements, muscle tone, and deep tendon reflexes. The overall score ranges from 0 to 78. Scores >74 for children >18 months of age and >73 for children between 12 and 18 months of age indicate adequate neurological development.

### Neurosensory evaluation

We performed auditory evaluations with the Simonek hearing kit [23], an adapted conditioned play audiometry test, used to screen children up to 48 months of age. It was performed by speech therapists and physicians trained to the assessment. It includes eight musical objects with different frequency levels from 38.2 to 95.1 decibels. The kit evaluates reflexes, attention, location, orientation in front of different auditory stimuli, and otoacoustic emissions (OAE), which are used for the detection of auditory changes of cochlear origin. The ophthalmologic evaluation included an appropriately focused physical exam, external ocular examination, ocular biomicroscopy, and indirect ophthalmoscopy with pupillary dilation, which has been used to evaluate CZS-associated abnormalities [24].

### Statistical analysis

We used the statistical package SPSS v21 for data analysis. We used frequency and percentages to characterize categorical data and median and interquartile range to characterize quantitative data. We grouped participants by seroconversion status during pregnancy. We compared developmental and clinical changes between these two groups using the Mann-Whitney U test for continuous variables and Fisher’s exact test for categorical variables. We measured associations between ZIKV exposure during pregnancy and study outcomes by Relative Risk (RR) with a 95% confidence interval (95% CI). To calculate the PAF of abnormalities secondary to intrauterine ZIKV exposure, we used the formula PAF = P_e_ (RR − 1) / [P_e_ (RR– 1) + 1], where P_e_ is the proportion of the population that was exposed. The 95% CI was calculated by substitution: PAF lower limit = P_e_ (RR_L_− 1) / [P_e_ (RR_L_− 1) + 1] and PAF upper limit = P_e_ (RR_U_− 1) / [P_e_ (RR_U_− 1) + 1], where RR_L_ and RR_U_ are the lower and upper limits of the 95% CI of RR, respectively [25].

## Results

From January 2015 to December 2016, we identified 66/2016 (3.3%) pregnant women within the cohort to potentially include in our analysis. Of these, 18 were not included: 15 due to change in residence to a household outside the study site, and 3 due to refusal to participate. Among the 48 (72.7%) women who consented to participate, there were 49 reported pregnancies, including 46 live births, 2 miscarriages and 1 stillbirth (Fig 1).

### Sociodemographic characteristics and exposure to ZIKV

**Fig 2:**
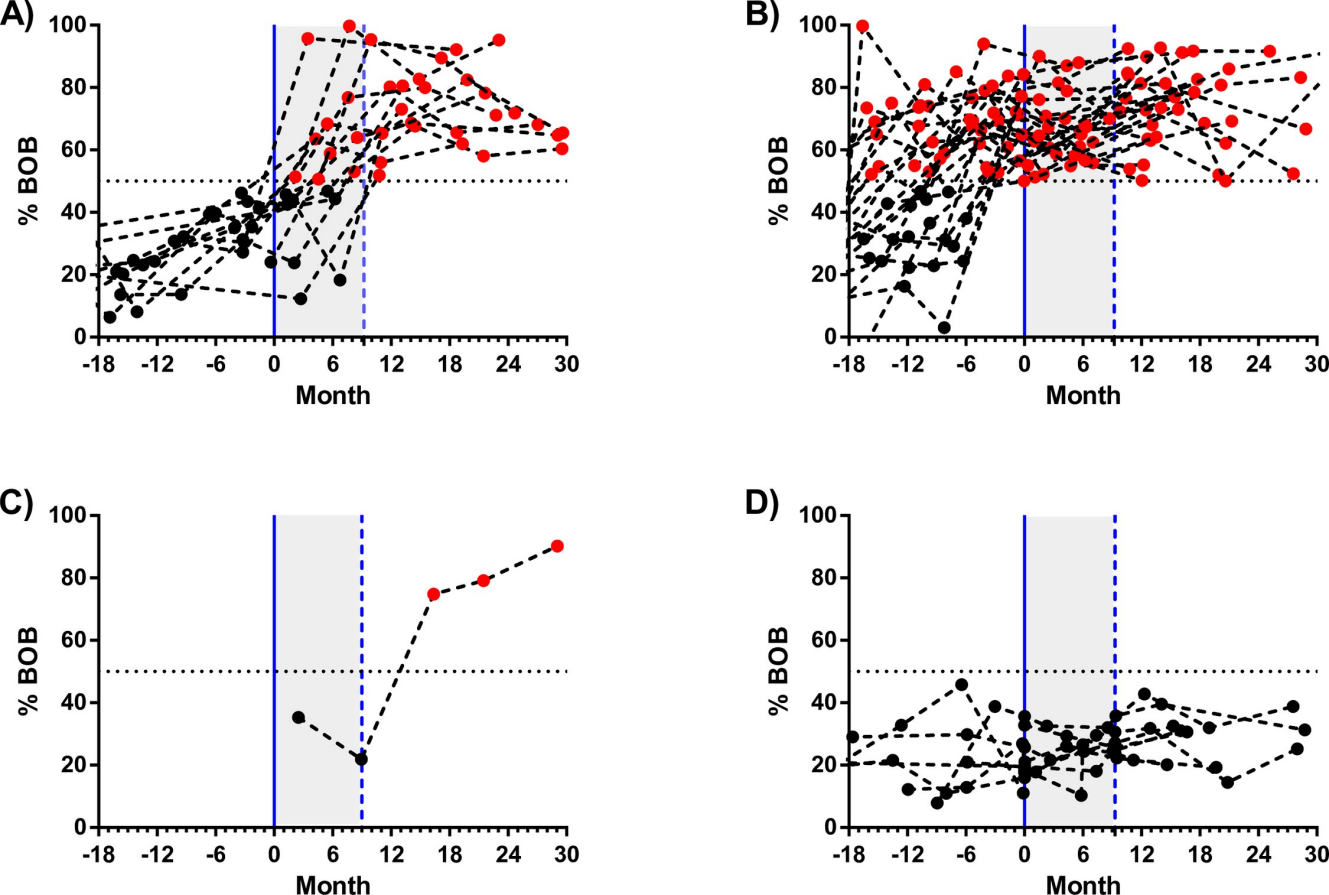
Timeline of follow-up standardized by conception day and ZIKV serological status of mothers. The continuous blue line represents estimated conception, calculated as the difference between date of birth and the gestational age at birth. The dashed blue line represents day of birth and the grey area between them represents the pregnancy period. Black and red dots are ZIKV BOB-negative and BOB-positive samples, respectively. (A) Mothers with evidence of probable ZIKV infection during the pregnancy period, (B) mothers with evidence of ZIKV infection before the pregnancy period, (C) mothers with evidence of ZIKV infection after the pregnancy period, and (D) mothers without evidence of ZIKV infection during the follow-up.

Demographic and socioeconomic information and history of pregnancy are described in Table 1. Most mothers were young, self-identified as black, and completed elementary school. 39 (84.8%) of the women had had ZIKV infection at some point during the study period: 13 (28.2%) seroconverted during pregnancy, 25 (54.3%) before pregnancy, and one (2.2%) after pregnancy (Table 1 and Fig 2). Out of the 13 who seroconverted during pregnancy, nine were considered as confirmed and four as probable. The difference between the first (negative) and the second (positive) samples was 6.4 months (IQR 5.6–7.2) (S1 Table).

**Table 1:** Baseline demographic and clinical characteristics of mothers in the pregnancy cohort—Salvador, Brazil, January 2015 to December 2016.

Characteristics	ZIKV exposed* No. (%) or median (IQR) (N = 13)	ZIKV unexposed^†^ No. (%) or median (IQR) (N = 33)	p-value
**Median mother age, median (IQR), years**	25 (21–31)	25 (20–30)	0.98
**Black ethnicity**^‡^	7 (53.8%)	22 (66.7%)	0.63
**Lower educational level**^‡^	5 (38.5%)	10 (30.3%)	0.84
**Previous pregnancies**			
0	3 (23.1%)	10 (30.3%)	0.79
1–3	6 (46.2%)	16 (48.5%)	0.87
≥ 4	4 (30.8%)	7 (21.2%)	
**Medical history**			
Arterial hypertension	0 (0.0%)	3 (9.1%)	0.72
Osteoarthritis	0 (0.0%)	1 (3.0%)	0.99
Prior sexually transmitted infection	1 (7.7%)	1 (3.0%)	0.99
**Health problems during pregnancy**			
Urinary infection	5 (38.5%)	9 (27.3%)	0.68
Vaginal fungus infection	2 (15.4%)	4 (12.1%)	0.99
TORCH infections	0 (0.0%)	0 (0.0%)	NA
HIV infection	0 (0.0%)	0 (0.0%)	NA
**Symptoms during pregnancy**			
Fever	4 (30.8%)	6 (18.2%)	0.57
Rash	1 (7.7%)	3 (9.1%)	0.99
Conjunctival injection	0 (0.0%)	0 (0.0%)	NA
Myalgia and arthralgia	1 (7.7%)	0 (0.0%)	0.99
Headache	3 (23.1%)	3 (9.1%)	0.42
**Median of prenatal care, median (IQR)**	4 (2–8)	6 (4–8)	0.28
**Median of prenatal ultrasonography, median (IQR)**	3 (2–4)	3 (2–4)	0.82
**Prenatal ultrasonography with malformations**	0 (0.0%)	0 (0.0%)	NA
**Nutritional supplement use**			
Ferrous sulphate	10 (76.9%)	28 (84.8%)	0.80
Folic acid	8 (61.5%)	24 (72.7%)	0.48
Other vitamins	2 (15.4%)	6 (18.2%)	0.99
**Caesarean delivery**	9 (69.2%)	24 (72.7%)	0.99
**Birth characteristics**			
Gestational age at birth, median (IQR) weeks	40 (39–40)	40 (39–40)	0.82
Preterm birth	1 (7.7%)	0 (0.0%)	0.26
Full term	11 (84.6%)	33 (100.0%)	
Postterm birth	1 (7.7%)	0 (0.0%)	0.26
**Mothers’ ZIKV NS1 BOB ELISA result**			
Positive	13 (100.0%)	26 (78.8%)	NA
During pregnancy	13 (100.0%)	0 (0.0%)	NA
Before pregnancy	0 (0.0%)	25 (75.8%)	NA
After pregnancy	0 (0.0%)	1 (3.0%)	NA
Negative	0 (0.0%)	7 (21.2%)	NA

* ZIKV-exposed mothers with seroconversion to ZIKV during pregnancy.

† Unexposed: mothers who were not infected during pregnancy (without seroconversion or seroconversion before or after pregnancy).

‡ Black ethnicity was compared with White (n = 3) and Mixed ethnicities (n = 14). Lower educational level was defined as no education or only up to first or second grade completed.

TORCH, Toxoplasmosis, Rubella, Cytomegalovirus, Herpes infections and Others.

NA, not applicable.

ZIKV NS1 BOB, ZIKV nonstructural protein 1 blockade-of-binding ELISA.

### Anthropometric and neurodevelopment outcomes

**Fig 3:**
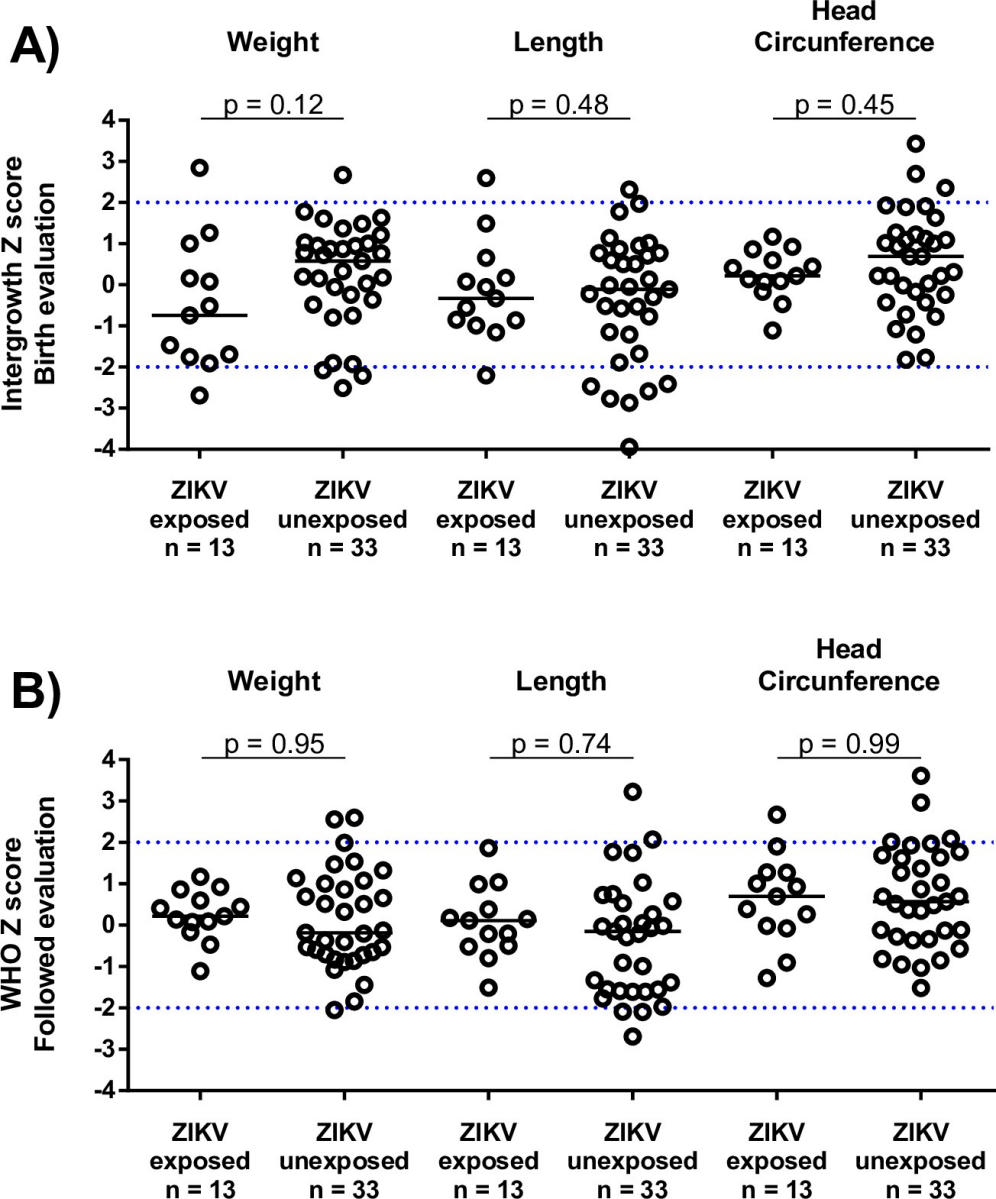
Anthropometric and neurodevelopment outcomes in children with and without evidence of ZIKV seroconversion during their mothers’ pregnancies. (A) Anthropometric measures at birth using Intergrowth parameters and (B) anthropometric measures during the follow-up period using WHO parameters.

We did not identify severe alterations in the anthropometric and neurological evaluations by Hammersmith Infant Neurological Examination (HINE) (Fig 3, Table 2, and S2 Table). Sixteen children (34.8%) were classified as at risk or emerging in the Bayley screening evaluation, which led to follow-up evaluation with the complete Bayley III (S1 Fig). We found scores equal to or below 85 (<-1 SD) in 6 children in the cognitive domain (13%), 5 in language (10%), and 2 in motor (4%). Our results show that children of mothers with ZIKV seroconversion during pregnancy, compared to children of mothers who did not seroconvert, had a 5.1-fold (95%CI 1.1–24.4) higher risk of a Bayley III score 1SD below the mean (≤85) in the cognitive domain, with a PAF of 53.5% (95%CI 1.4–86.9). There was no statistically significant difference in risk with respect to the language and motor development (Table 2) or socio-emotional and adaptive domains (S3 Table).

**Table 2:** Neurological examination, ophthalmological, auditory and neurodevelopmental evaluation outcomes.

Outcomes	ZIKV-exposed No. (%) or median (IQR)	ZIKV-unexposed No. (%) or median (IQR)	RR (IC 95%) or p-value*	PAF (95% CI)
**Neurological examination**				
Child age in months	24 (20–27)	17 (14–26)	0.18*	NA
Median HINE total score (IQR)	75 (74–77)	76 (74–78)	0.77*	NA
HINE total score >74	13/13 (100.0%)	33/33 (100)	NA	NA
**Ophthalmologic alteration**^‡^	1/9 (11.1%)	2/25 (8.0%)	1.39 (0.14–13.53)	9.3 (-29.5–76.8)
Child age in months	24 (20–26)	17 (14–28)	0.24*	NA
Cataract	0/9 (0.0%)	1/25 (4.0%)		
Ptosis	1/9 (11.1%)	0/25 (0.0%)		
Synechiae	0/9 (0.0%)	1/25 (4.0%)		
**Auditory evaluation**^‡^				
Child age in months	24 (20–27)	18 (14–29)	0.24*	NA
Abnormal auditory behavior test	5/13 (38.5%)	2/31 (6.5%)	5.96 (1.32–26.89)	59.5 (8.6–88.4)
Abnormal OAE	0/6 (0.0%)	0/15 (0.0%)	NA	NA
**Neurodevelopmental function**^**†**^				
Child age in months	21 (17–24)	15 (12–23)	0.18*	NA
**Bayley-III screening evaluation**				
At risk	1/13 (7.7%)	1/33 (3.0%)	2.56 (0.18–36.62)	30.4 (-30.0–90.9)
Emerging	4/13 (30.8%)	10/33 (30.3%)	1.07 (0.41–2.76)	1.8 (-5.7–13.8)
Competent	8/13 (61.5%)	22/33 (66.7%)	1	
**Bayley-III complete evaluation ≤-1 SD (at least one standard deviation)**	4/13 (30.8%)	7/33 (21.2%)	1.45 (0.51–4.14)	11.3 (-16.1–49.1)
Cognitive score ≤-1 SD	4/13 (30.8%)	2/33 (6.1%)	5.07 (1.05–24.44)	53.5 (1.4–86.9)
Language score ≤-1 SD	1/13 (7.7%)	4/33 (12.1%)	0.63 (0.07–5.16)	-10.3 (-35.7–54.0)
Motor score ≤-1 SD	1/13 (7.7%)	1/33 (3.0%)	2.54 (0.17–37.63)	30.3 (-30.6–91.2)

* Mann Whitney test p-value.

† Infants classified as at risk and emerging in Bayley screening were revaluated using a complete. protocol of Bayley. Children classified as competent in Bayley screening were considered as >-1 SD in the Cognitive, Language and Motor scores of the complete Bayley protocol.

‡ Nine of the ZIKV exposed and 25 of the ZIKV unexposed children completed the ophthalmological evaluation; 31 of the ZIKV unexposed children completed the auditory behavior test; and six of the ZIKV exposed and 15 of the ZIKV unexposed children completed the OAE test.

Bayley-III, Bayley Scales of Infant and Toddler Development, Third edition.

HINE, Hammersmith Infant Neurological Examination.

OEA, otoacoustic emissions test.

NA, not applicable.

SD, standard deviation.

IQR, interquartile ratio.

PAF, population attributable fraction.

### Neurosensory outcomes

We performed ophthalmological evaluations on 34 children. Abnormal findings included a single instance each of unilateral cataract (2.9%), unilateral ptosis (2.9%), and posterior synechiae (2.9%). We evaluated 21 children using an OAE test and found no abnormalities. In the auditory behavioral evaluation, 7 (15.9%) showed behavioral problems related to orientation, localization, and attention in response to an auditory stimulus. Auditory behavioral assessment also showed that children born to women with ZIKV seroconversion during pregnancy had a risk of abnormal auditory behavior 6.0-fold (95% CI 1.3–26.9) higher than that of those born to women without seroconversion, with a PAF of 59.5% (95% CI 8.6–88.4) (Table 2).

We also compared children of women with symptomatic and asymptomatic ZIKV infection during pregnancy and found no significant difference in clinical and developmental outcomes (S4 Table). This was also true when comparing children of mothers with confirmed and probable intrauterine ZIKV exposure. Moreover, the statistical associations between intrauterine ZIKV exposure and cognitive and auditory behavioral abnormalities persisted after excluding probable exposed cases from the analysis (S5 Table).

## Discussion

Our findings from a cohort of women living in a slum community in Brazil suggest that both symptomatic and asymptomatic ZIKV infection during pregnancy is associated with mild neurodevelopmental abnormalities in congenitally infected children without apparent birth defects. Children of mothers exposed to ZIKV during pregnancy had 5-fold greater risk of mild cognitive delay (30.8% of the cohort) than unexposed children, as well as a 6-fold greater risk of abnormal auditory behavior. Furthermore, more than half of these abnormalities could be attributed to intrauterine ZIKV exposure. Although we did not identify differences in the global neurodevelopmental (considering the overall Bayley scale) between children born to women with and without ZIVK infection during the pregnancy, 30.8% of the exposed children had mild cognitive impairment (Bayley cognitive scale). Among them, approximately half could be attributed to in-utero ZIKV infection (~15% of the cohort).

In Brazil, during the ZIKV epidemic, we found evidence that more than half of the cases of children with neurodevelopmental delay in an urban slum community may be attributable to intrauterine ZIKV exposure. Our population-based study, including a control group of children from the same community, gave us the unique opportunity to calculate the percentage of abnormalities attributable to ZIKV intrauterine exposure. We found no statistically significant difference in risk of impairment with respect to maternal symptomology during pregnancy, suggesting that a considerable group of children born to asymptomatic ZIKV(+) mothers may be experiencing neurodevelopmental delay without prior suspicion. A previous study of pre-school children from middle- and low-income countries found cognitive delay in 10.1% of children, attributing 2.5 to42.7% of the delay in these children to social determinants such as poverty, low levels of stimulation in the home, and did not have access to unimproved drinking water and sanitation [26]. We attributed 53.5% of the mild cognitive delay and 59.5% of auditory behavioral abnormalities in our cohort to ZIKV infection during pregnancy, which are higher than that attributed to any sole socio-environmental factor described in the study above. The combination of intrauterine ZIKV exposure and socio-environmental factors may all influence the extent of delay and could be an interesting line of future enquiry. However, there was no significant difference in the other neurodevelopmental domains (language, motor, social-emotional, and adaptive behavior Bayley scale scores) and the overall Bayley score between exposed and unexposed children.

Our findings did not reveal any children with severe cognitive delay but rather a greater frequency of mild cognitive delay (30.8%) in this Brazilian slum community. These results are similar to other recent findings with subtle differences. A study in the U.S. Territories and Freely Associated States showed that amongst 1,495 children exposed to ZIKV, 9% had neurodevelopmental abnormalities possibly associated with CZS [3]. In another cohort study of 146 children in Rio de Janeiro, 28.1% had mild delay in some neurodevelopmental domain [10]. In the Rio de Janeiro cohort, 4.1% and 5.5% of children without microcephaly from symptomatic pregnancies had mild and severe cognitive delay, respectively [10]. Another study of children with intrauterine ZIKV exposure in Colombia and the U.S. did not find developmental impairments but demonstrated decreasing developmental trajectories in different skills, including communication, social cognition, and mobility [12].

With respect to language and motor development, we found a proportion of mild delay in each group (<12%), but without significant difference between the exposed and control groups. In contrast, the Rio cohort had higher proportions of children who experienced mild language delay (28.7%), severe language delay (12.3%), and mild motor delay (16.4%), though without a control group for comparison [10]. Another Brazilian study followed children born from symptomatic pregnancies without microcephaly at one year of age, finding that 12.5% of them (7/56) had neurodevelopmental delay including language, motor, or behavioral deficits [9].

It is possible that other types of impairments may appear in children exposed to ZIKV in-utero, as has been the case for other viruses. Vertical transmission of HIV, for instance, has been associated with mild and severe cognitive, motor, and language impairments [27,28], moreover the prevalence of these manifestations increase with child age, during the development [29]; West Nile Virus has long been associated with encephalitis, meningitis, or possible neurodevelopmental disorders [8]; and cytomegalovirus, rubella, toxoplasmosis, and herpes virus infections have been associated with cognitive deficits, language development, and visual changes [7]. These infections have also been related to hearing loss, detected even after birth and the neonatal period, and which can be progressive and fluctuating [1,30]. Although the children in our study did not experience hearing loss, we reported behavioral auditory problems in 38% of children, related to orientation, localization, and attention to auditory stimuli. Our results add to the body of knowledge of neurodevelopmental sequelae in children with in utero ZIKV exposure, but it is necessary to consider that other manifestations may arise later on and in different contexts. Still, our results reinforce that monitoring developmental impairments in the preschool period is critical for this pediatric population, as sustained delay without intervention has increased risk of learning and behavioral problems and functional impairments in the next stage of life [27,31].

We acknowledge our study’s limitations. First, the sample size in this population study was limited, affecting the study’s statistical power, as reflected in the wide ranges in the confidence intervals of relative risks describing the associations between ZIKV exposure and auditory and cognitive delays. Second, despite multiple attempts, there was difficulty in moving participants to a hospital to collect neuroimaging and complete ophthalmological and auditory evaluations, thus possibly affecting the rate of defects that could have been identified. Furthermore, otoacoustic emissions test is not a preferred methodology for acoustic evaluation; however, it can help to screen and rule out auditory problems to be confirmed later by Auditory Brainstem Response (ABR) [32]. Moreover, serological surveys do not allow for exact estimation of asymptomatic infection time, as we classified four cases as probable seroconversion during pregnancy; however, our conclusions still held after exclusion of probable exposed cases from the analysis. Cross-reactivity between ZIKV and DENV is also of concern in serological studies, especially considering that >90% of the population (≥20 years old) in this community had DENV antibodies before the Zika epidemic [14]. However, 96% of samples collected prior to the epidemic were negative for ZIKV antibodies, suggesting lack of cross-reactivity between viruses. The high ZIKV attack rate in this study (>70%), similar to previous results from the same area [14], and the persistence of high antibody levels after seroconversion across subsequent time points, suggest that the majority of seroconversions over this period could be attributed to ZIKV infection. Additionally, while molecular confirmation is ideal in the clinical setting, it is not a practical method to characterize ZIKV exposure in populations, since only 34% of infections in this population were associated with fever or rash [14]. Thus, reliance on this method could lead to underestimation of infection in pregnant women and the incidence of sequelae in their children.

In summary, we documented a high incidence of mild neurodevelopmental impairments, specifically in the cognitive domain and auditory behavior, that could be attributable to both symptomatic and asymptomatic ZIKV intrauterine exposure. Children with neurodevelopmental impairment benefit from early intervention, behooving healthcare providers and caregivers to be alert to these potential clinical manifestations and perform timely detection of developmental and behavioral problems. These early interventions should also include children with mild cognitive impairment born to asymptomatic mothers during pregnancy, who may have a different perception of risk to their children’s development, and thus should prompt widespread screening of potential ZIKV infection during the epidemic period as part of pregnancy care protocols.

## Supporting information

S1 Table Serum samples collection period among 13 ZIKV-exposed (confirmed and probable) children. (DOCX)

S2 Table Anthropometric evaluation outcomes in children with and without evidence of ZIKV infection in their mothers during pregnancy. (DOCX)

S3 Table Neurodevelopmental outcomes of 16 children with abnormal neurodevelopmental screening result. (DOCX)

S4 Table Cognitive development and auditory behavior of 13 children born to women exposed to ZIKV during pregnancy, classified by presence or absence symptoms during pregnancy. (DOCX)

S5 Table Neurological examination, ophthalmological, auditory and neurodevelopment evaluation outcomes in ZIKV-exposed (confirmed and probable) and unexposed children. (DOCX)

S1 Fig Bayley III score of 16 children without normal neurodevelopmental screening result. (TIF)
