# Supplementary material for: Developmental outcomes in children exposed to Zika virus in utero from a Brazilian urban slum cohort study
Source: PLoS Negl Trop Dis. 2021 Feb 5;15(2):e0009162. doi: 10.1371/journal.pntd.0009162 (PMC7891708; doi:10.1371/journal.pntd.0009162)
Supplement: S1 Table — (DOCX) [file pntd.0009162.s001.docx]

**S1 Table:** Serum samples collection period among 13 ZIKV-exposed (confirmed and probable) children.

|  | Mediam (IQR) |
| --- | --- |
| **Time between sample collections in month n= 13** | 6.4 (5.6 – 7.2) |
| **ZIKV seroconversion during the pregnancy n=9** |  |
| First (negative) sample collection, pregnancy month | 2.1 (1.2 – 2.7) |
| Second (positive) sample collection, pregnancy month | 8.2 (7.6 – 9) |
| **Provable ZIKV seroconversion during the pregnancy n=4** |  |
| First (negative) sample collection, pregnancy month | 6 (5.6 – 6.5) |
| Second (positive) sample collection, month after the birth of | 3.5 (2.5 – 4.1) |

IQR: interquartile range
