# Supplementary material for: Developmental outcomes in children exposed to Zika virus in utero from a Brazilian urban slum cohort study
Source: PLoS Negl Trop Dis. 2021 Feb 5;15(2):e0009162. doi: 10.1371/journal.pntd.0009162 (PMC7891708; doi:10.1371/journal.pntd.0009162)
Supplement: S2 Table — (DOCX) [file pntd.0009162.s002.docx]

**S2 Table.** Anthropometric evaluation outcomes in children with and without evidence of ZIKV infection in their mothers during pregnancy.

| **Characteristics** | **Exposed median (IQR)**  **(N=13)** | **Unexposed median (IQR)**  **(N=33)** | **p-value** |
| --- | --- | --- | --- |
| **Birth evaluation** |  |  |  |
| **Median sore Z***(IQR) |  |  |  |
| Weight | -0.7 (-1.8 - 0.2) | 0.6 (-0.4 - 1.0) | 0.12 |
| Length | -0.3 (-0.9 - 0.2) | -0.1 (-1.2 - 0.8) | 0.48 |
| Head circumference | 0.2 (0.1 - 0.6) | 0.7 (-0.2 - 1.2) | 0.35 |
| **Follow-up evaluation** |  |  |  |
| Median Children age, months (IQR) | 18 (14-22) | 11 (8 – 20) | 0.19 |
| **Median score Z**†(IQR) |  |  |  |
| Weight | 0.1 (-0.5 - 0.4) | -0.2 (-0.7 - 1.0) | 0.95 |
| Length | -0.1 (-0.8 - 0.4) | -0.2 (-1.6 - 0.6) | 0.74 |
| Head circumference | 0.7 (-0.02 - 1.3) | 0.6 (-0.3 - 1.6) | 0.99 |

* Intergrowth parameters^19^

† WHO parameters^20^

IQR, interquartile ratio
