# Supplementary material for: Developmental outcomes in children exposed to Zika virus in utero from a Brazilian urban slum cohort study
Source: PLoS Negl Trop Dis. 2021 Feb 5;15(2):e0009162. doi: 10.1371/journal.pntd.0009162 (PMC7891708; doi:10.1371/journal.pntd.0009162)
Supplement: S3 Table — (DOCX) [file pntd.0009162.s003.docx]

**S3 Table.** Neurodevelopmental outcomes of 16 children with abnormal neurodevelopmental screening result.

| **Bayley scales** | **Exposed**  **median (IQR)**  **(N=5)** | **Unexposed**  **median (IQR)(N=11)** | **p-value** |
| --- | --- | --- | --- |
| **Median percentile scale score (IQR)** |  |  |  |
| Cognitive | 9.0 (9 – 9) | 37 (25 – 63) | 0.019 |
| Language | 18.0 (18 – 27) | 27 (13 – 42) | 0.913 |
| Motor | 58.0 (27 – 75) | 50 (42 – 68) | 0.743 |
| Social – Emotional scale | 25.0 (16 – 63) | 9 (5 – 63) | 0.221 |
| Adaptive behavior | 42.0 (30 – 47) | 39 (4 – 55) | 0.743 |

IQR, interquartile ratio
