# Supplementary material for: Developmental outcomes in children exposed to Zika virus in utero from a Brazilian urban slum cohort study
Source: PLoS Negl Trop Dis. 2021 Feb 5;15(2):e0009162. doi: 10.1371/journal.pntd.0009162 (PMC7891708; doi:10.1371/journal.pntd.0009162)
Supplement: S4 Table — (DOCX) [file pntd.0009162.s004.docx]

**S4 Table.** Cognitive development and auditory behavior of 13 children born to women exposed to ZIKV during pregnancy, classified by presence or absence symptoms during pregnancy.

|  | **Symptomatic***  **No. (%) or median (IQR)**  n **= 4** | **Asymptomatic**  **No. (%) or median (IQR)**  n **= 9** | **p value** |
| --- | --- | --- | --- |
| **Median HINE total score (IQR)** | 74 (74 – 75) | 76 (75 – 78) | 0.11 |
| **Birth evaluation** |  |  |  |
| **Median score Z**†(IQR) |  |  |  |
| Weight | -0.86 (1.08–0.71) | -0.3 (-0.9–0.1) | 0.71 |
| Length | -1.33 (0.6 – 0.7) | -1.5 (-1.8–0.1) | 0.71 |
| Head circumference | -0.04 (1.0– 0.6) | 0.2 (0.1–0.4) | 0.60 |
| **Follow-up evaluation** |  |  |  |
| **Median score Z**‡(IQR) |  |  |  |
| Weight | -1.0 (0.1 – 0.3) | 0.2 (-0.2–1.0) | 0.26 |
| Length | -0.4 (0.2 – 0.8) | -0.1 (-0.9–0.5) | 0.82 |
| Head circumference | -0.3 (0.8 – 0.4) | 1.0 (0.0–1.3) | 0.41 |
| **Neurodevelopmental function** |  |  |  |
| **Cognitive domain≤-1SD** | 1 (25.0%) | 3 (33.3%) | 0.99 |
| **Language domain≤-1SD** | 1 (25.0%) | (0.0%) | 0.61 |
| **Motor domain≤-1SD** | 1 (25.0%) | (0.0%) | 0.61 |
| **Auditory behavior test abnormal** | 1 (25.0%) | 4 (44.4%) | 0.97 |

^*^ Symptomatic was define as the presence of fever, rash, myalgia or arthralgia during pregnancy

† Intergrowth parameters^19^

‡ WHOparameters^20^

IQR, interquartile ratio
