# Supplementary material for: Developmental outcomes in children exposed to Zika virus in utero from a Brazilian urban slum cohort study
Source: PLoS Negl Trop Dis. 2021 Feb 5;15(2):e0009162. doi: 10.1371/journal.pntd.0009162 (PMC7891708; doi:10.1371/journal.pntd.0009162)
Supplement: S5 Table — (DOCX) [file pntd.0009162.s005.docx]

**S5Table.** Neurological examination, ophthalmological, auditory and neurodevelopment evaluation outcomes in ZIKV-exposed (confirmed and probable) and unexposed children.

|  | **ZIKV exposed** | | **ZIKV- unexposed**  **(N=33)** |
| --- | --- | --- | --- |
| **Outcomes** | **Confirmed**  **(N=9)** | **Probable**  **(N=4)** |  |
| **Neurological examination** |  |  |  |
| Median HINE total score (IQR) | 76(75–78) | 74(74–75.5) | 76.0 (74–78) |
| Total score >74 | 9/9(100%) | 4/4(100%) | 13/33 (100%) |
| **Ophthalmologic alteration** | 1/6(16.7%) | 0/4 (0.0%) | 2/25 (8.0%) |
| Cataract | 0/6 (0.0%) | 0/4 (0.0%) | 1/25 (4.0%) |
| Ptosis | 1/6 (16.7%) | 0/4 (0.0%) | 0/25 (0.0%) |
| Synechiae | 0/6 (0.0%) | 0/4 (0.0%) | 1/25 (4.0%) |
| **Auditory evaluation** |  |  |  |
| Abnormal auditory behavior test | 4/9(44.4%)* | 1/4 (25.0%) | 2/25 (6.5%)* |
| Abnormal OAE | 0/3 (0.0%) | 0/3 (0.0%) | 0/15 (0.0%) |
| **Neurodevelopmental function†** |  |  |  |
| **Bayley-III screening evaluation** |  |  |  |
| At risk | 1/9(11.1%) | 0/4 (0.0%) | 1/33 (3.0%) |
| Emerging | 2/9(22.2%) | 1/4 (25.0%) | 10/33 (30.3%) |
| Competent | 5/9 (55.6%) | 3/4 (75.0%) | 22/33 (66.7%) |
| **Bayley-III complete evaluation ≤-1SD** | 4/9 (44.4%) | 0/4 (0.0%) | 7/33 (21.2%) |
| **Cognitive score ≤-1SD** | 4/9 (44.4%)* | 0/4 (0.0%) | 2/33 (6.1%)* |
| **Language score ≤-1SD** | 1/9 (11.1%) | 0/4 (0.0%) | 4/33 (12.1%) |
| **Motor score ≤-1SD** | 1/9 (11.1%) | 0/4 (0.0%) | 1/33 (3.0%) |

* Fisher test p-value<0.05

† Infants classified as at risk and emerging in Bayley screening were revaluated using a complete protocol of Bayley. Children classified as competent in Bayley screening were considered as having >-1SD in the Cognitive, Language and Motor scores of the complete Bayley protocol.

Bayley-III, Bayley Scales of Infant and Toddler Development, Third edition.

HINE, Hammersmith infant neurological examination.

OEA, otoacoustic emissions test.

NA, not applicable.

SD, standard deviation.

IQR, interquartile ratio.
