# Supplementary figures and images for: Developmental outcomes in children exposed to Zika virus in utero from a Brazilian urban slum cohort study
Source: PLoS Negl Trop Dis. 2021 Feb 5;15(2):e0009162. doi: 10.1371/journal.pntd.0009162 (PMC7891708; doi:10.1371/journal.pntd.0009162)

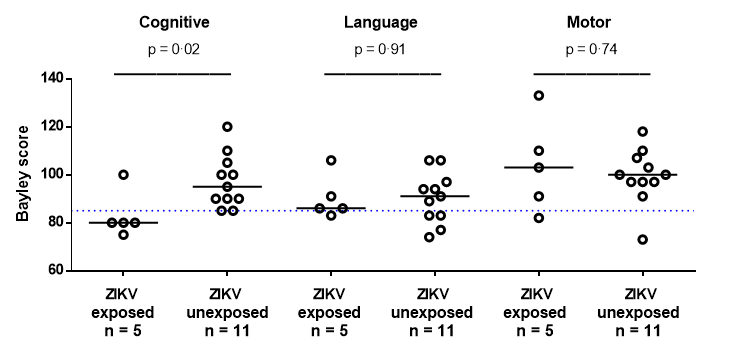

Supplement: S1 Fig — (TIF) [file pntd.0009162.s006.tif]
